# Characterization of fatty acid modifying enzyme activity in staphylococcal mastitis isolates and other bacteria

**DOI:** 10.1186/1756-0500-5-323

**Published:** 2012-06-22

**Authors:** Thea Lu, Joo Youn Park, Kelleen Parnell, Larry K Fox, Mark A McGuire

**Affiliations:** 1 Department of Biological Sciences, University of Idaho, Moscow, USA; 2 Department of Animal and Veterinary Sciences, University of Idaho, Moscow, USA; 3 Department of Veterinary Clinical Science, Washington State University, Pullman, USA; 4 Department of Basic Sciences, Mississippi State University, Mississippi State, USA

**Keywords:** Fatty acid modifying enzyme, Lipase, Coagulase-negative staphylococci

## Abstract

**Background:**

Fatty acid modifying enzyme (FAME) has been shown to modify free fatty acids to alleviate their bactericidal effect by esterifying fatty acids to cholesterol or alcohols. Although it has been shown in previous studies that FAME is required for *Staphylococcus aureus* survival in skin abscesses, FAME is poorly studied compared to other virulence factors. FAME activity had also been detected in coagulase-negative staphylococci (CNS). However, FAME activity was only surveyed after a bacterial culture was grown for 24 h. Therefore if FAME activity was earlier in the growth phase, it would not have been detected by the assay and those strains would have been labeled as FAME negative.

**Results:**

Fifty CNS bovine mastitis isolates and several *S. aureus, Escherichia coli*, and *Streptococcus uberis* strains were assayed for FAME activity over 24 h. FAME activity was detected in 54% of CNS and 80% *S. aureus* strains surveyed but none in *E. coli* or *S. uberis*. While some CNS strains produced FAME activity comparable to the lab strain of *S. aureus*, the pattern of FAME activity varied among strains and across species of staphylococci. All CNS that produced FAME activity also exhibited lipase activity. Lipase activity relative to colony forming units of these CNS decreased over the 24 h growth period. No relationship was observed between somatic cell count in the milk and FAME activity in CNS.

**Conclusions:**

Some staphylococcal species surveyed produced FAME activity, but *E. coli* and *S. uberis* strains did not. All FAME producing CNS exhibited lipase activity which may indicate that both these enzymes work in concert to alter fatty acids in the bacterial environment.

## Background

**Figure 1:**
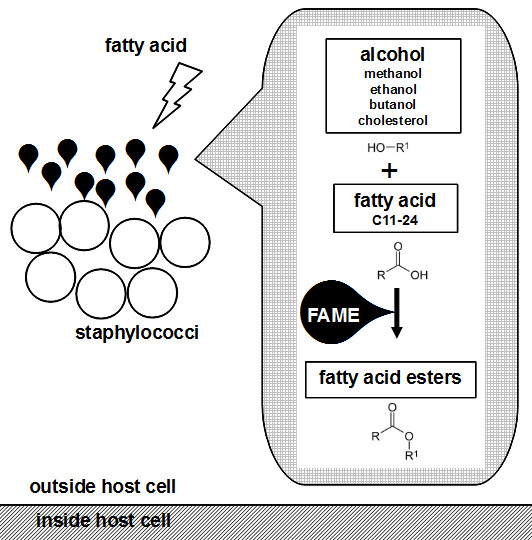
**Esterification of fatty acids by fatty acid modifying enzyme (FAME).** Staphylococci (open circles) colonize the host and block the bactericidal effects of fatty acids (open bolt) by secreting FAME (solid balloons) which esterifies fatty acids to alcohol esters.

Fatty acid modifying enzyme (FAME) was first described by Mortensen *et al.*[1] while studying abscesses caused by *Staphylococcus aureus*. It was found that culture filtrates of *S. aureus* contained an extracellular enzyme that counteracted the bactericidal activity of lipids within abscesses. When fatty acid samples were incubated with FAME in the presence of ethanol, ethyl esters were produced. The FAME enzyme acts by esterifying free fatty acids to short chain primary alcohols and cholesterol, with cholesterol being the preferred substrate [1] (Figure 1). While FAME activity can be detected experimentally, the FAME protein and its corresponding gene have yet to be identified.

In an abscess, microbicidal fatty acids are part of the first line of defense for the host against invading pathogens [2]. Staphylococcal FAME, however, is inhibited by glycerides and it is thought that to alleviate this inhibition, *S. aureus* also produces lipase which liberates the free fatty acids from the glyceride backbone so that FAME can further esterify the free fatty acids [3]. Previous studies have shown that about 80% of the staphylococcal strains that produce lipase also produce FAME. Strains that did not produce both of these enzymes were more sensitive to free fatty acids. Long and colleagues [4] speculated that survival in abscesses and pathogenesis would require these two enzymes.

Previously, FAME activity was assayed in a selected number of other staphylococci including coagulase-negative staphylococci (CNS). However, FAME activity was only surveyed after CNS were grown for 24 h [4] and in one strain of *S. epidermidis* for up to 12 h [5]. Therefore if FAME activity occurred earlier in the growth phase but the enzyme producing this activity was degraded before the assayed time point, it would not have been detected by the assay and those strains would have been labeled as FAME negative. *S. aureus* strains that had been previously studied included the lab strain *S. aureus* RN6390 and clinical isolates found in intraperitoneal abscesses [1,2]. However, staphylococci clinically important in bovine mastitis have yet to be characterized for FAME activity. We hypothesized that FAME activity is dependent on staphylococcal strain and is related to virulence.

## Results

Fifty CNS strains isolated from bovine milk were assayed for FAME activity over 24 h. Bacterial growth assessed by optical density (OD) and colony forming units (CFU) were found to be similar in all strains within species. In our growth conditions, cultures reached stationary phase after 8–12 h. FAME activity was detected in at least one strain of each CNS species except for *Staphylococcus equorum*, *Staphylococcus gallinarum*, and *Staphylococcus sciuri*. Out of the 50 CNS strains surveyed, 27 (54%) strains had detectable FAME activity. Bovine mastitis isolates of *Escherichia coli* and *Streptococcus uberis* did not produce detectable FAME activity over 24 h of growth. All *S. aureus* strains surveyed except one produced detectable FAME activity (Table 1).

**Table 1:** FAME activity in tested bacteria

**Species**	**No. of strains tested**	**No. of strains with FAME activity (%)**	**Relative range of FAME activity at 25 h**
*S. aureus*	6	5 (83)	0–187^*^
*Escherichia coli*	2	0 (0)	0
*Streptococcus uberis*	1	0 (0)	0
**Coagulase Negative Staphylococci**			
*S. capitis*	1	1 (100)	19–47^*^
*S. caprae*	3	3(100)	13–42^*^
*S. chromogenes*	15	4(27)	0–22^*^
*S. epidermidis*	2	2 (100)	4–6^*^
*S. equorum*	1	0 (0)	0
*S. gallinarum*	1	0 (0)	0
*S. haemolyticus*	8	4 (50)	0–21^*^
*S. hominis*	1	1 (100)	1–10^*^
*S. hyicus*	3	2 (67)	0–7^*^
*S. sciuri*	2	0 (0)	0
*S. simulans*	5	5 (100)	10–35^*^
*S. succinus*	2	1 (50)	0–5^*^
*S. xylosus*	6	4 (67)	0–172^*^
Total CNS	50	27 (54)	0–172

^*^Percent esterification compared to a standard with significant activity *P* < 0.05 from negative control.

**Figure 2:**
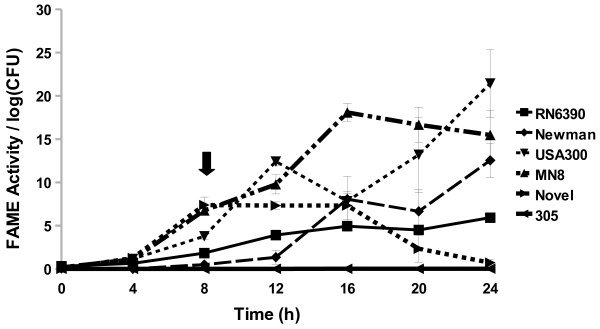
**Most surveyed*S. aureus*strains produce FAME activity.***S. aureus* strains RN6390, Newman, USA300, MN8, Novel, and Newbould 305 were assayed for FAME activity/log CFU over 24 h. CFUs were generally the same among the strains with the beginning of stationary phase indicated by the arrow. Results are the average of three experiments. Vertical bars represent standard error of the observation; if no bar is apparent the standard error is smaller than the symbol. Detectable activity for RN6390, Newman, USA300, MN8, and Novel was seen at 8 h (*P* < 0.05).

Different *S. aureus* strains exhibited different patterns of FAME activity. In the laboratory strain *S. aureus* RN6390, FAME activity increased with bacterial growth; activity reached a plateau by 16 h at 5% esterification of oleic acid to butyl oleate compared to a standard of butyl stearate (FAME activity) per log CFU and remained stable for the rest of stationary phase to 24 h (Figure 2). Both *S. aureus* Newman and *S. aureus* USA300 reached maximal FAME activity at 24 h (12.5% and 21% FAME activity/log CFU, respectively). *S. aureus* MN8 peaked at 16 h with 18% esterification per log CFU. The bovine mastitis isolate *S. aureus* Novel reached a maximum FAME activity of 7% esterification per log CFU at 8 h, but the activity decreased at 20 h with very little activity (0.7%) detected at 24 h. The other bovine mastitis isolate tested, *S. aureus* Newbould 305, did not have any detectable FAME activity over 24 h.

**Figure 3:**
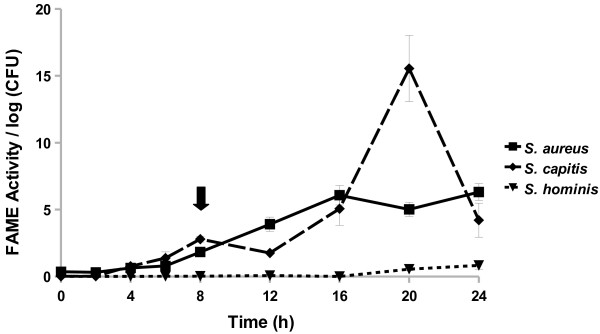
***S. aureus, S. capitis*, and*S. hominis*exhibit different patterns FAME activity.***S. aureus* RN6390, *S. capitis*, and *S. hominis* were assayed for FAME activity/log CFU over 24 h. CFUs were generally the same among the strains with the beginning of stationary phase indicated by the arrow. Results are the average of three experiments. Vertical bars represent standard error of the observation; if no bar is apparent the standard error is smaller than the symbol. Detectable activity for *S. aureus* and *S. capitis* was seen at 4 h and for *S. hominis* at 24 h (*P* < 0.05).

The pattern of FAME activity over a 24 h period exhibited by CNS was different among species and strains within species. FAME activity increased in *Staphylococcus capitis* throughout its growth and peaked in stationary phase at 20 h (Figure 3). *S. capitis* produced appreciable FAME activity from 16 to 24 h with 15% esterification per log CFU at 20 h. By contrast, *Staphylococcus hominis* FAME activity did not rise above 0.5% at any point during the 24 h culture.

**Figure 4:**
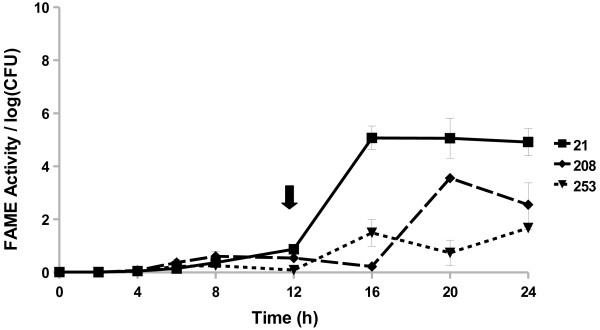
***S. caprae*produces FAME activity during stationary phase.***S. caprae* isolates were assayed for FAME activity/log CFU over 24 h. CFUs were generally the same among the strains with the beginning of stationary phase indicated by the arrow. Results are the average of three experiments. Vertical bars represent standard error of the observation; if no bar is apparent the standard error is smaller than the symbol. Detectable activity for all *S. caprae* isolates was seen at 8 h (*P* < 0.05).

**Figure 5:**
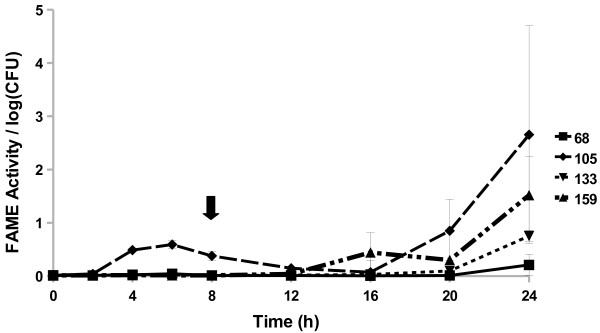
***S. chromogenes*isolates exhibit different patterns of FAME activity.***S. chromogenes* isolates were assayed for FAME activity/log CFU over 24 h. CFUs were generally the same among the strains with the beginning of stationary phase indicated by the arrow. Results are the average of three experiments. Vertical bars represent standard error of the observation; if no bar is apparent the standard error is smaller than the symbol. *S. chromogenes* isolates had detectable FAME activity at 24 h (*P* < 0.05).

**Figure 6:**
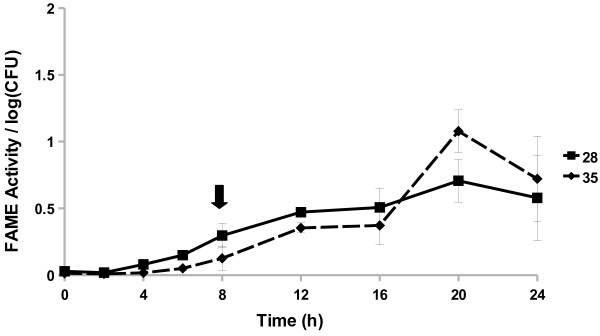
**Two*S. epidermidis*isolates increase FAME activity over 24 h.***S. epidermidis* isolates were assayed for FAME activity/log CFU over 24 h. CFUs were generally the same among the strains with the beginning of stationary phase indicated by the arrow. Results are the average of three experiments. Vertical bars represent standard error of the observation; if no bar is apparent the standard error is smaller than the symbol. FAME activity for all *S. epidermidis* isolates was detected at 6 h (*P* < 0.05).

**Figure 7:**
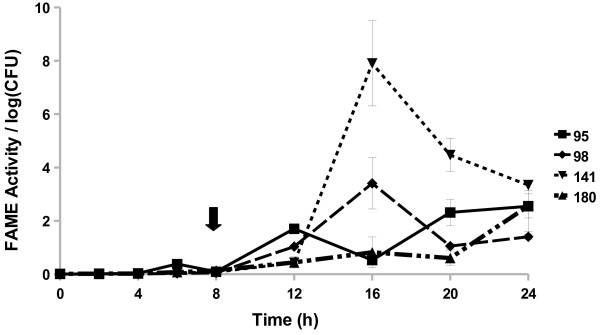
*** S. haemolyticus*isolates exhibit different patterns of FAME activity.***S. haemolyticus* isolates were assayed for FAME activity/log CFU over 24 h. CFUs were generally the same among the strains with the beginning of stationary phase indicated by the arrow. Results are the average of three experiments. Vertical bars represent standard error of the observation; if no bar is apparent the standard error is smaller than the symbol. FAME activity for all *S. haemolyticus* isolates was detected at 12 h (*P* < 0.05).

**Figure 8:**
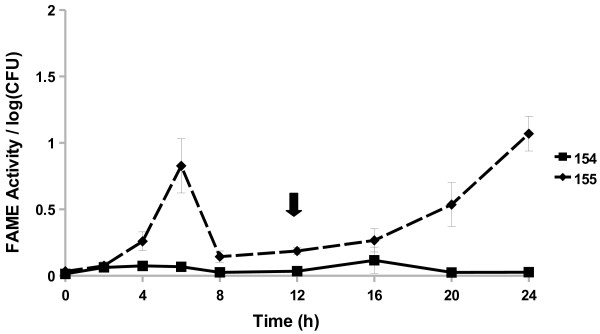
***S. hyicus*produces low FAME activity over 24 h.***S. hyicus* isolates were assayed for FAME activity/log CFU over 24 h. CFUs were generally the same among the strains with the beginning of stationary phase indicated by the arrow. Results are the average of three experiments. Vertical bars represent standard error of the observation; if no bar is apparent the standard error is smaller than the symbol. Both isolates had detectable activity at 4 h (*P* < 0.05).

**Figure 9:**
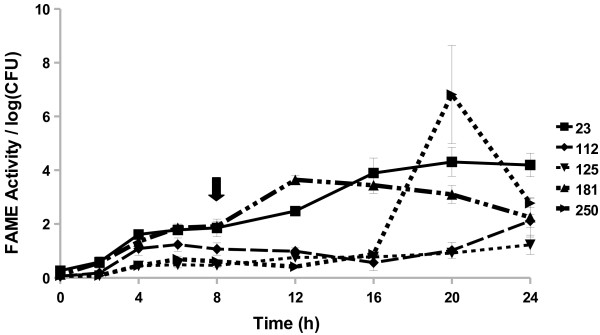
***S. simulans*isolates exhibit a range of patterns for FAME activity.*** S. simulans* isolates were assayed for FAME activity/log CFU over 24 h. CFUs were generally the same among the strains with the beginning of stationary phase indicated by the arrow. Results are the average of three experiments. Vertical bars represent standard error of the observation; if no bar is apparent the standard error is smaller than the symbol. FAME activity for all *S. simulans* isolates was detected at 4 h (*P* < 0.05).

**Figure 10:**
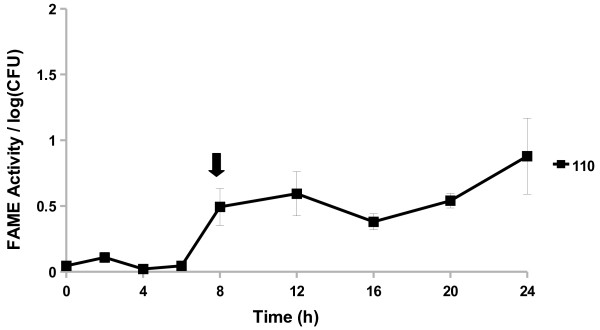
***S. succinus*produces low FAME activity over 24 h.** Two *S. succinus* isolates were assayed but only one had detectable FAME activity. The FAME producing strain is shown for FAME activity/log CFU over 24 h. CFUs were generally the same among the strains with the beginning of stationary phase indicated by the arrow. Results are the average of three experiments. Vertical bars represent standard error of the observation; if no bar is apparent the standard error is smaller than the symbol. FAME activity for *S. succinus* was detected at 8 h (*P* < 0.05).

**Figure 11:**
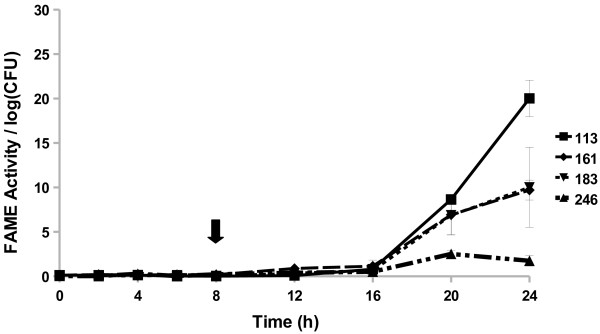
***S. xylosus*produces the highest FAME activity of all surveyed CNS strains.***S. xylosus* isolates were assayed for FAME activity/log CFU over 24 h. CFUs were generally the same among the strains with the beginning of stationary phase indicated by the arrow. Results are the average of three experiments. Vertical bars represent standard error of the observation; if no bar is apparent the standard error is smaller than the symbol. *S. xylosus* isolates had detectable FAME activity at 20 h (*P* < 0.05).

All *Staphylococcus caprae* strains examined started producing FAME activity by 8 h (Figure 4). The three strains reached a maximum activity by 16, 20, or 24 h. The *S. caprae* strains surveyed had FAME activity peaking around 1.7–5% by 24 h. Four out of 15 *Staphylococcus chromogenes* strains produced FAME activity; activity was greatest 20–24 h in culture (up to 2.7% esterification) with one strain also producing some activity (0.6% esterification per log CFU) from 4 to 8 h (Figure 5). All *Staphylococcus epidermidis* (Figure 6) strains surveyed produced increasing FAME activity up to 20 h of culture with maximum activity less than 1% esterification per log CFU. Four *Staphylococcus haemolyticus* strains had no detectable FAME activity, however, four other strains produced detectable FAME activity beginning at 12 h (Figure 7). *S. haemolyticus* strains exhibited considerable variation in the pattern of FAME activity with maximal activity at different times of culture and ranging from 2.5 to 8% (Figure 8). One strain of *Staphylococcus hyicus* produced less than 0.5% FAME activity per log CFU over the entire growth period with just above the detectable limit at 16 h, while another strain produced a detectable amount of FAME activity at 4 h with activity increasing after 16 h up to 1% at 24 h (Figure 8). The FAME producing strains of *Staphylococcus simulans* showed several patterns of FAME activity throughout culture (Figure 9). These *S. simulans* strains started producing detectable FAME activity at 4 h. Two strains produced relatively consistent FAME activity (1–2%) between 4 and 24 h. Two other strains of *S. simulans* had FAME activity increase above 3% between 12 or 16 h. One strain of *S. simulans* had peak FAME activity of 6.8% at 20 h. At 24 h, FAME activity in *S. simulans* strains ranged from 1 to 4%. One strain of *Staphylococcus succinus* (Figure 10) started producing detectable FAME activity at 8 h but activity never rose above 0.1%. *Staphylococcus xylosus* was the CNS strain which produced the most FAME activity. In the four *S. xylosus* strains with FAME activity, activity was detected at 20 h (Figure 11). At 24 h, FAME activity in these *S. xylosus* strains ranged from 1.7 to 20% esterification per log CFU.

Lipase activity per CFU was compared to FAME activity per CFU over 24 h in 27 CNS strains with FAME activity. All CNS strains surveyed produced lipase activity. The strains showed decreased lipase activity (7.11 ± 0.52 log of percent lipase activity divided by CFU; mean ± SD) at 24 h compared to 0 h. CNS strains that exhibited no detectable FAME activity produced a range of lipase activity (4.35 ± 4.81 log of percent lipase activity divided by CFU; mean ± SD). In strains that did produce FAME activity, the relationship between FAME and lipase activity was poor (r^2^ = 0.1) when activity for each were considered at each individual time point. However, the relationship of the mean FAME and lipase activities of all CNS with FAME activity at each time point correlated well (r^2^ = 0.92). Lipase activity per CFU decreased over the 24 h growth period.

**Figure 12:**
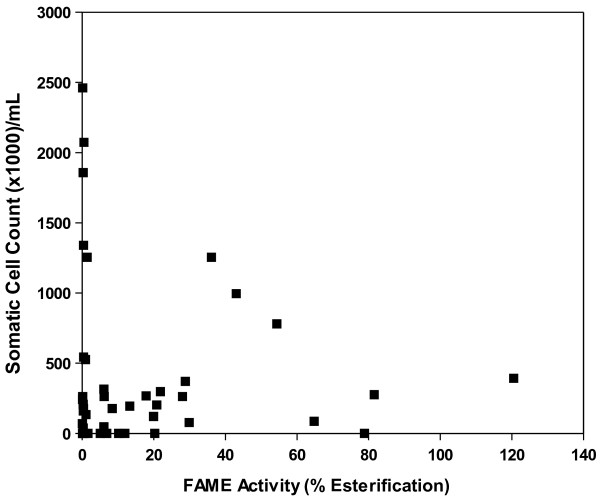
**Staphylococci with FAME activity are compared to somatic cell counts in milk from commercial dairy cows.** Somatic cell counts from the milk samples from which the CNS strains were isolated are compared with FAME activity assayed from 24 h culture supernatants. No clear relationship was observed between FAME activity and SCC in milk for all CNS strains (r^2^ = 0.01).

No clear relationship was observed between FAME activity and somatic cell count (SCC) in milk for all CNS strains at 24 h (Figure 12). CNS strains with FAME activity less than 30%, 30–60%, and greater than 60% had SCC (mean x 1,000 per mL ± SD) of 331 ± 585.2, 1,010.1 ± 237.4, and 150.9 ± 175.8, respectively.

## Discussion

*Staphylococcus aureus, S. epidermidis*, and several other staphylococcal species have been shown to possess FAME activity [4], but this enzyme activity had not been assayed over a 24 h growth period or in other bacteria. We surveyed FAME activity in CNS isolated from bovine milk, *S. aureus* clinical isolates, and *E. coli* and *S. uberis* bovine mastitis isolates which served as negative controls and compared it to a laboratory strain of *S. aureus*. Using a quantitative assay of FAME activity, we found that the relative range of FAME activity varied across and within staphylococcal species over a 24 h growth period. No FAME activity was found in *E. coli* or *S. uberis*. In *S. aureus*, the highest FAME activity was found in strains originally isolated from human infections, *S. aureus* USA300, *S. aureus* MN8, and *S. aureus* Newman. *S. aureus* Novel and *S. aureus* Newbould 305, bovine mastitis isolates which cause acute and chronic disease respectively [6], produced different patterns of FAME activity. There was also a difference in virulence between strains of *S. aureus* differing in FAME activity as tested in a murine model; *S. aureus* strains with FAME activity were able to persist longer in intraperitoneal abscesses than strains that did not produce this activity [1]. The differences in FAME activity observed in *S. aureus* Novel and *S. aureus* Newbould 305 and the severity of disease caused by each strain suggest that FAME may play a role in modulating virulence in bovine mastitis. Contrary to data collected by Long *et al.*[4], we found that some *S. haemolyticus* strains produced FAME activity. FAME activity varied in CNS; some did not rise above ~10% esterification at any point during the growth curve while others produced an appreciable amount or similar pattern of FAME activity like *S. aureus*. There were others such as *S. equorumS. gallinarum*, and *S. sciuri* which produced no detectable FAME activity. However, this may not indicate that these species lack FAME activity, only that the isolates tested did not produce this activity under our assay conditions.

For the FAME positive CNS strains, FAME production peaked at different stages of growth. The discrepancy of FAME activity between different strains as well as species may be due to strain differences. The *agr* locus has high genetic variability in *Staphylococcus* species which would lead to a variation in regulation of any downstream staphylococcal products [7]. FAME activity has been shown to be regulated by *agr* and *sar,* global regulators known to modulate the production of virulence factors such as exotoxins [8,9]. Other regulators may also play a part in differences of FAME production in each strain. *S. aureus* RN6390, for example, is a natural deletion mutant of *rsbU*, a regulator of σ^B^, which controls bacterial response to acid stress [10]. The potential of *S. aureus* and CNS to cause a variety of diseases suggests a complex mechanism for pathogenesis which includes the regulatory input of environmental and host signals. The expression of staphylococcal virulence genes *in vivo* may also depend on distinct host signals from the target tissue [9]. Considering strain variability of *agr* alone, every strain could regulate FAME differently. As a result, FAME may not necessarily be produced by *Staphylococcus* spp. at a particular time point or growth condition. In our assay of several time points throughout a 24 h growth period, we found several *Staphylococcus* spp. exhibited maximum FAME production during time points earlier than 24 h.

Lipase activity is produced by a group of enzymes found in a wide number of both Gram-negative and Gram-positive bacteria [11]. FAME has been hypothesized to work in concert with lipase to modify antimicrobial fatty acids in the host [4]. Kapral *et al.*[3] noted that FAME activity is optimal within abscesses at pH 6 while lipase activity was optimal at pH 8. But more recent biochemical analyses reveal that some staphylococcal lipases are active over a broad pH range including pH 6 [11]. In our study, all strains observed with FAME activity also exhibited lipase activity lending support to this hypothesis that the presence of both these activities are required for fatty acid modification in the bacterial environment. We found however that CNS strains without FAME activity also had lipase activity. These bacteria were originally isolated from bovine milk where lipids are abundant [12,13]. Although lipase liberates potentially bactericidal fatty acids, the lipase-producing bacteria in milk may not be lipolytically active because most of the milk lipids are sequestered in fat globules [27]. A small increase in free fatty acids, however, is found in milk of cows with clinical mastitis [28]. Since staphylococci produce several lipases of which only a few have been characterized [11], the conditions in milk may not be optimal for lipase production in the CNS strains surveyed in this study. In CNS that did produce FAME activity, lipase and FAME activity per CFU decreased as bacterial growth progressed over 24 h although these enzyme activities increased in the overall culture.

As one of the first lines of defense, the host recruits innate immune cells, such as neutrophils or polymorphonuclear leukocytes, to the site of inflammation [14]. Somatic cell count (SCC) in milk is used as a reliable indicator of inflammation in the mammary gland since it consists primarily of neutrophils and other leukocytes [15]. Previous observations suggested that FAME had a possible role in suppressing the host immune response [2]. However staphylococci associated with lower SCC varied in FAME production, and those with very high somatic cell counts (above 1,000,000 cells/mL) had less than 60% FAME activity (Figure 12). This lack of association between FAME activity and SCC indicates that FAME in CNS plays little role in modulating the recruitment of immune cells but does not eliminate other possible functions. Modification of host lipids can also aid pathogenesis by disrupting host membranes, interfering with host signaling, and providing nutrients for the bacterium. In order to modify these lipids, pathogens can produce an array of enzymes that act on these substrates. Lipases have been shown to aid pathogens in immune evasion. Staphylococcal lipase decreases phagocytosis and intracellular killing by human granulocytes [16]. These lipases may also be involved in liberating host cell lipids for nutrient uptake [17]. Cholesterol is the preferred substrate for FAME [1] thus previously identified FAME negative bacteria could have been incorrectly characterized due to current methods which use butanol instead of cholesterol. Cholesterol can be manipulated by pathogens to influence host cell membrane fluidity, signaling pathways, and lipid raft composition. *Helicobacter pylori* modifies cholesterol to escape the immune system by inhibiting phagocytosis and T-cell activation [18]. Staphylococci may be using FAME for immunomodulation in a similar way by altering cholesterol homeostasis.

Bacteria with properties that make them impermeable to these antimicrobial lipids, such as thicker cell walls, may not require an enzyme like FAME [19]. However, FAME activity has only been surveyed in staphylococcal strains, *E. coli*, and *S. uberis* so it is unknown whether FAME is also widely found among bacterial species or is a uniquely staphylococcal enzyme. We have also shown that an assessment of FAME activity cannot rely solely on one time point since activity did not occur only during the stationary growth phase. Instead, FAME activity patterns vary across strains and species. To determine if FAME is unique to staphylococci, a comprehensive survey including more species and a variety of growth conditions would be required.

## Conclusions

Some staphylococcal species surveyed produced FAME activity, but *E. coli* and *S. uberis* strains did not, suggesting that FAME activity may be limited to some staphylococcal species. For the FAME positive CNS strains, FAME production peaked at different stages of growth. The greatest FAME activity was found in strains originally isolated from human infections. *S. aureus* Novel, a strain associated with acute disease, produced detectable FAME activity while *S. aureus* Newbould 305, a strain of chronic disease, did not. The severity of disease caused by each of strain suggests that FAME may play a role in modulating virulence in bovine mastitis. All FAME producing CNS exhibited lipase activity which may indicate that both these enzymes work in concert to alter fatty acids in the bacterial environment.

## Methods

### Bacterial strains

The strains used in this study are listed in Table 2. The CNS strains were initially isolated from milk samples collected from cows with intramammary infection in dairies in Idaho and Washington and identified by 16 S ribosomal DNA sequencing [11]. Each strain was streaked on tryptic soy agar (TSA) from glycerol stocks and incubated overnight at 37°C before inoculating into 3 mL tryptic soy broth (TSB) and grown in a shaker incubator at 37°C and 250 rpm for 16 h. To determine the growth of each strain, the 16 h culture was inoculated into TSB at 1:100 in triplicate and grown at 37°C with shaking at 250 rpm for 24 h. Every 2 h from 0 to 8 h and every 4 h from 8 to 24 h, growth of bacterial culture was determined by assaying optical density (OD) at 600 nm and by plating serial dilutions on TSA to determine colony forming units (CFU) for each strain in triplicate. Plates were incubated at 37°C for 15 h before determining bacterial growth.

**Table 2:** List of strains used in this study

**Species**	**Strain**	**Source or reference**
*Escherichia coli*	TOP10	Invitrogen, Carlsbad, CA
*Escherichia coli*	ATCC25922	ATCC25922
*Streptococcus uberis*	ATCC27958	ATCC27958
*S. aureus*	RN6390	NCTC 8325-4^*^
*S. aureus*	MN8	[20]^*^
*S. aureus*	Newman	[21]^*^
*S. aureus*	USA300	[22]^*^
*S. aureus*	Novel	[23]
*S. aureus*	Newbould 305	ATCC29740
*S. capitis*	240	[12]
*S. caprae*	21	[12]
*S. caprae*	208	[12]
*S. caprae*	253	[12]
*S. chromogenes*	1	[12]
*S. chromogenes*	14	[12]
*S. chromogenes*	61	[12]
*S. chromogenes*	68	[12]
*S. chromogenes*	74	[12]
*S. chromogenes*	100	[12]
*S. chromogenes*	105	[12]
*S. chromogenes*	111	[12]
*S. chromogenes*	133	[12]
*S. chromogenes*	136	[12]
*S. chromogenes*	159	[12]
*S. chromogenes*	184	[12]
*S. chromogenes*	207	[12]
*S. chromogenes*	232	[12]
*S. chromogenes*	267	[12]
*S. epidermidis*	28	[12]
*S. epidermidis*	35	[12]
*S. equorum*	117	[12]
*S. gallinarum*	196	[12]
*S. haemolyticus*	20	[12]
*S. haemolyticus*	39	[12]
*S. haemolyticus*	95	[12]
*S. haemolyticus*	98	[12]
*S. haemolyticus*	141	[12]
*S. haemolyticus*	180	[12]
*S. haemolyticus*	247	[12]
*S. haemolyticus*	275	[12]
*S. hominis*	260	[12]
*S. hyicus*	120	[12]
*S. hyicus*	154	[12]
*S. hyicus*	155	[12]
*S. sciuri*	71	[12]
*S. sciuri*	99	[12]
*S. simulans*	23	[12]
*S. simulans*	112	[12]
*S. simulans*	125	[12]
*S. simulans*	181	[12]
*S. simulans*	250	[12]
*S. succinus*	261	[12]
*S. succinus*	110	[12]
*S. xylosus*	101	[12]
*S. xylosus*	113	[12]
*S. xylosus*	161	[12]
*S. xylosus*	183	[12]
*S. xylosus*	200	[12]
*S. xylosus*	246	[12]

^*^kindly supplied by Drs. G.A. Bohach and K.S. Seo (Mississippi State University, Mississippi).

### FAME activity assay

Bacterial cultures were pelleted at 12,000 x *g* for 2 min, and supernatant was removed for analysis. Activity of FAME was determined by combining 700 μL of 0.1 M sodium phosphate buffer (pH = 6) with 250 μL of culture supernatant and 50 μL oleic acid (4 mg/mL butanol) and incubating the solution for 20 min at 37°C with gentle shaking at 100 rpm. The reaction was terminated immediately by adding 19 mL chloroform:methanol (2:1 v/v). An internal standard containing butyl stearate (100 μL added from stock of 0.5 mg/mL of chloroform) was used to quantify the amount of oleic acid esterified to butyl oleate. Lipids were extracted using the Folch procedure [24] and butyl esters quantified. Samples were analyzed using gas chromatography (Agilent Technologies 6890 N GC, California) equipped with a 30 m x 0.25 mm with 0.15 μm film DB-17HT capillary column (Agilent J&W Scientific, California) with programmed temperature runs using hydrogen as the carrier gas. The oven temperature was initially 100°C and then increased 1.5°C/min until 165°C and then held constant for 45 min. The temperature was then ramped at 30°C/min until 350°C. Injection port temperature was at 200°C. One μL of each sample was injected and the split ratio was 5:1 with flow rate held constant at 5 mL/min. Butyl oleate and butyl stearate (internal standard) peaks were detected with a flame ionization detector identified using standards and the area of the peaks quantified and used to calculate the percent of oleic acid esterified in each sample. Controls included TSB with no added bacteria in place of culture supernatant, and the phosphate buffer alone plus butanol and oleic acid to ensure the product did not form spontaneously.

### Lipase assay

Lipase activity at each time point was determined by adding 50 μl bacterial supernatant to 1 mL of lipase assay reagent (10 mM para-nitrophenyl palmitate, 0.1% Triton X-100 in ethanol) [25]. The reaction was incubated at 37°C, 300 rpm for 40 min. Absorbance was measured at 405 nm on a Wallac Victor2 plate reader (PerkinElmer, Turku, Finland). A standard curve was developed using dilutions of a supernatant from a *S. aureus* RN6390 24 h culture.

### Somatic Cell Count

Milk samples at room temperature within 4 h post-collection were used. SCC was determined in triplicate using the DeLaval cell counter (DeLaval International AB, Tumba, Sweden) [26]. A cassette containing propidium iodide as the fluorescent stain was used to collect 60 μl milk. The sample was exposed to an LED light source that causes the cell nuclei to give fluorescent signals which were subsequently recorded as SCC in milk.

### Statistics

Data were analyzed by Student’s *t*-test, SAS 9.2, to determine the threshold of FAME activity and lipase activity compared with negative controls. Significant and detectable activity was declared when FAME or lipase activity was different (*P* < 0.05) from both negative controls.

## Abbreviations

CFU, Colony forming units; CNS, Coagulase-negative staphylococci; FAME, Fatty acid modifying enzyme; OD, Optical density; SCC, Somatic cell count; SD, Standard deviation; TSA, Tryptic soy agar; TSB, Tryptic soy broth.

## Competing interests

The authors declare that they have no competing interests.

## Authors’ contributions

TL and MAM conceived the study. JYP and LKF identified and provided the CNS strains used in this study. TL, JYP, and KP carried out the experiments. TL analyzed the results. TL and MAM drafted the manuscript. Additionally, all authors have read and approved the final manuscript.
